# Feeding increases the number of offspring but decreases parental investment of Red Sea coral *Stylophora pistillata*

**DOI:** 10.1002/ece3.5712

**Published:** 2019-10-02

**Authors:** Jessica Bellworthy, Jorge E. Spangenberg, Maoz Fine

**Affiliations:** ^1^ The Mina and Everard Goodman Faculty of Life Sciences Bar Ilan University Ramat Gan Israel; ^2^ The Interuniversity Institute for Marine Sciences in Eilat Eilat Israel; ^3^ Institute of Earth Surface Dynamics (IDYST) University of Lausanne Lausanne Switzerland

**Keywords:** climate change, coral, diet, lipids, parental investment, Red Sea

## Abstract

1. Successful reproductive output and recruitment is crucial to coral persistence and recovery following anthropogenic stress. Feeding is known to alter coral physiology and increase resilience to bleaching.
2. The goal of the study was to address the knowledge gap of the influence of feeding on reproductive output and offspring phenotype.
3. Colonies of *Stylophora pistillata* from the Northern Gulf of Aqaba (Red Sea) were fed an *Artemia* diet or unfed for 5 months during gametogenesis, fertilization, and brooding. In addition, time to settlement and mortality of planulae were assessed at water temperatures ranging from winter temperature (22°C) to three degrees above average peak summer temperature (31°C). A range of physiological parameters was measured in parents and offspring.
4. In brooding parents, feeding significantly increased protein concentration and more than tripled the number of released planulae. Planulae from unfed colonies had higher chlorophyll per symbiont concentration and concomitantly higher photosynthetic efficiency compared to planulae from fed parents. In settlement assays, planulae showed a similar thermal resistance as known for this Red Sea adult population. Mortality was greater in planulae from unfed parents at ambient and 3°C above ambient temperature despite higher per offspring investment in terms of total fatty acid content. Fatty acid profiles and relative abundances were generally conserved between different fed and unfed colonies but planulae were enriched in monounsaturated fatty acids relative to adults, that is, 16:1, 18:1, 20:1, 22:1, and 24:1 isomers.
5. Ultimately the availability of zooplankton could influence population physiology and recruitment in corals.

## Graphical abstract

Colonies of reef building coral *Stylophora pistillata* from the Gulf of Aqaba were fed for 5 months leading up to the peak reproductive period. Fed colonies produced three times as many offspring but with lower individual protein and fatty acid content. Conversely, planulae from unfed colonies had higher photochemical efficiency and higher chlorophyll levels, but still higher total mortality. Ultimately the availability of zooplankton could influence population physiology and recruitment in corals.

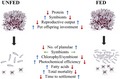

## 1 INTRODUCTION

Carry over or transgenerational effects occur when an offspring phenotype is influenced by more than just genes (Marshall & Uller, 2007) but also by the environmental experience of previous generation(s). These effects are one of the most important influences on offspring phenotype (Mousseau, 2002). In adverse environmental conditions, carry over effects may be expressed as a decrease in offspring quality or fitness (Bernardo, 2015) and may only emerge after multigenerational exposure (Gibbin et al., 2017). Conversely, an adjustment to offspring quality, even as a result of environmental stress, may serve to increase progenies' likelihood to survive and is in such cases viewed as adaptive (Mousseau, 2002; Mousseau & Dingle, 1991). For example, parental exposure to future ocean conditions enabled complete acclimation of aerobic scope in offspring of a tropical fish (Donelson, Munday, McCormick, & Pitcher, 2012) and metabolic acclimation of larvae after just 6 weeks' parental exposure in a brooding reef‐building coral (Putnam & Gates, 2015).

To date, multigenerational experiments are largely limited to organisms with short generation times. However, in a rapidly changing climate, the ability for longer‐lived species' adaptation to keep pace has come into question. For example, reef‐building corals are the foundation organisms of the coral reef ecosystem and yet their persistence is threatened by the environmental consequences of increasing global carbon dioxide levels. Experimental studies of carry over effects, which manipulate the parental environment, are few in scleractinian corals (Bellworthy, Menoud, Krueger, Meibom, & Fine, 2019; Putnam & Gates, 2015; Putnam, Ritson‐Williams, Cruz, Davidson, & Gates, 2018). This is likely attributed to corals' relatively long time to sexual maturity, extended gametogenesis period, as well as typically a single annual reproductive cycle, and an inability to reliably induce spawning ex situ (but see Craggs et al., 2017). Carry over effects have been observed in a single study using brooding reef‐building coral, *Pocillopora damicornis*, after just 6 weeks of parental exposure to elevated temperature and ocean acidification (Putnam & Gates, 2015); planulae from exposed parents displayed metabolic acclimation to future ocean conditions, whereas planulae from control parents did not (Putnam & Gates, 2015).

Reproductive output is strongly influenced by parental condition in corals. Following severe bleaching on the Great Barrier Reef in 1998, 45% of colonies of the scleractinian coral *Acropora hyacinthus* were gravid compared to 100% in the year previous to bleaching (Baird & Marshall, 2002). In addition, physical damage causes corals to significantly decrease reproductive output (Rinkevich & Loya, 1989; Van Veghel & Bak, 1994; Zakai, Levy, & Chadwick‐Furman, 2000). There therefore appears to be environmental constraints and an energetic trade‐off between parental recovery and reproductive output (Rinkevich, 1996). Importantly, the ability for coral populations to recover after disturbance events is strongly influenced by reproductive output and recruitment (Hughes et al., 2019) and the energy available for reproduction depends upon the nutritional status of the colony (both photosynthate translocation and feeding combined). However, the effect of parental diet upon reproductive output has not been tested in corals. Reproductive output is a combination of the number of offspring and the size of each individual or per offspring investment (POI). The quantity and quality of POI is particularly important in species with nonfeeding larvae (Marshall & Keough, 2007), since they must initially rely on these reserves for energy. To date, there has only been one study which directly tested for translocation of labeled carbon and nitrogen from vertically transmitted symbionts (i.e., given by the parent colony prior to release) to the host in coral planulae larvae (Kopp, Domart‐Coulon, Barthelemy, & Meibom, 2016). These authors concluded that while minimal translocation was observed, this was in quantities which would negligibly contribute to larval energetic demands (Kopp et al., 2016). Despite demonstrations of increased lipid utilization and decreased survival in darkness (Harii, Yamamoto, & Hoegh‐Guldberg, 2010), and changes in symbiont transcriptome upon larval infection (Mohamed et al., 2019), these data do not prove translocation per se and therefore a recent review of the topic concluded that during the larval phase a “mutualistic relationship cannot be unequivocally confirmed” (Mies, Sumida, Rädecker, & Voolstra, 2017). Additionally, there were no isotopic signatures of feeding found in planulae of Red Sea coral *Stylophora pistillata* (Alamaru, Yam, Shemesh, & Loya, 2009). Therefore, within the design and discussion of the following study, planulae are considered to be entirely lecithotrophic, neither feeding nor using photosynthetic metabolites.

*Stylophora pistillata* within the Gulf of Aqaba (GoA) and Northern Red Sea, along with multiple other scleractinian species from this region, is recognized as thermally resistant (Bellworthy & Fine, 2017; Fine, Gildor, & Genin, 2013; Osman et al., 2018). Over the past three decades, despite sea surface temperatures in the GoA rising at a rate faster than the global average (Krueger et al., 2017; Osman et al., 2018), the national monitoring program reports an increase in hard coral cover between 2004 and 2017 and no mass coral bleaching (Shaked & Genin, 2018). Likewise, experiments exposing adult *S. pistillata* to temperatures far beyond the assumed 28°C local bleaching threshold (1°C above maximum monthly mean temperature 26.75°C; Bellworthy & Fine, 2017) for up to 6 weeks did not result in any significant change in typical physiological indicators (Bellworthy & Fine, 2017; Fine et al., 2013; Krueger et al., 2017). A 5‐week parental exposure to ocean acidification (pH 7.6) and higher temperature (5°C above ambient) did not induce significant changes in either POI or offspring physiology, further attesting to the resistance of *S. pistillata* from the GoA both during early life stages and induction of carry over effects (Bellworthy et al., 2019). It has therefore been hypothesized that this region may act as a coral reef refuge from global climate change (Fine et al., 2013; but see Genevier, Jamil, Raitsos, Krokos, & Hoteit, 2019).

In the face of climate change, there are proposals to selectively breed adaptive, resistant corals (van Oppen et al., 2017; van Oppen, Oliver, Putnam, & Gates, 2015). Parental diet could potentially be used to improve POI and offspring fitness during early life history (McCormick, 2003). Feeding has been shown to significantly increase protein and chlorophyll *a* (Chl *a*) concentrations as well as photosynthetic and growth rates in adult *S. pistillata* (Borell, Yuliantri, Bischof, & Richter, 2008; Houlbreque, Tambutté, Allemand, & Ferrier‐Pagès, 2004; Houlbrèque, Tambutté, & Ferrier‐Pagès, 2003). Furthermore, enhanced feeding rates can increase resistance to and recovery from bleaching events (Grottoli, Rodrigues, & Palardy, 2006; Hughes & Grottoli, 2013; Tagliafico et al., 2017); however, the carry over effect of feeding has not been assessed in corals. Several questions can be identified, including (a) what are the effects of feeding on coral physiology and reproductive output, (b) is there a measurable carry over effect of parental diet to coral planulae, and (c) how do abrupt temperature changes—in light of global climate change—effect the settlement and mortality rates of planulae as a product of parental diet? These questions were addressed in this study with an experiment where *S. pistillata* colonies were fed for 5 months during gametogenesis, fertilization, and brooding, and the resulting effect on maternal condition, POI, and physiology were assessed. It was hypothesized that fed maternal colonies will produce planulae with greater POI but that this will not influence planulae thermal response in this thermally resistant population (Bellworthy & Fine, 2017; Bellworthy et al., 2019; Fine et al., 2013; Krueger et al., 2017). Time to settlement and survival of planulae were assessed under exposure to a broad thermal gradient ranging from 22 to 31°C where ambient seawater temperature was ca. 25°C. For the first time, we evaluate the effect of diet on the mother–offspring transfer of lipids—the dominant energetic substrate—in corals by comparison of the fatty acid profiles obtained by gas chromatography/mass spectrometry.

## 2 METHODS

### 2.1 Experimental conditions and collection of planulae

Mature colonies (*n* = 12) of *S. pistillata* (15–30 cm diameter) were collected from the field nursery located adjacent to the Interuniversity Institute of Marine Sciences in Eilat, Israel (IUI) at 8–12 m depth in early December 2015. Only colonies that appeared visually healthy, that is, no signs of disease, tissue loss or bleaching, were collected and maintained in ambient seawater at IUI. Colonies were placed directly into a flow through system consisting of two half pipe “troughs,” supplied constantly with filtered (500 µm mesh) natural seawater from an inlet at 30 m depth thereby matching seasonal temperature change. Above surface inflow (3 L/min) and overflow drainage provided aeration, water movement, and water turnover. Troughs are located outside and shaded by an overhead black mesh that reduces natural sunlight light levels to ca. 20% of ambient. Photosynthetically active radiation (PAR) levels under the shade were ca. 340–400 µmol m^−2^ s^−1^ during the experiment at midday on a cloudless day which corresponds to ca. 15 m water depth at the end of winter in the Gulf of Aqaba (Dishon, Dubinsky, Fine, & Iluz, 2012; Eyal et al., 2016), thereby approximating the light level at the colony collection depth. Furthermore, high light levels were avoided since the effects of feeding are more explicit under low light in corals (Ferrier‐Pagès, Hoogenboom, & Houlbrèque, 2011). Colonies were allocated to either fed or unfed treatment conditions in a way that resulted in a similar mean colony diameter per treatment. Fed colonies (*n* = 6) were initially fed once weekly for the first 8 weeks, and increased to twice weekly for a further 12 weeks until mid May 2016, for a total of 5 months. At dusk, incoming water flow was stopped for 30 min while 2 L of freshly hatched cultured *Artemia salina* nauplii were added to the tanks at a concentration of ca. 1 million individuals/L. Water movement was maintained by a power head pump (Aqua One Maxi 103, 1,200 L/hr). The remaining colonies (*n* = 6) were not fed zooplankton but had access to the typically low, oligotrophic ambient level nutrients from the GoA and the same light conditions as the fed group. Colonies were swapped between troughs once a month to reduce “tank effects.”

In order to facilitate planulae collection after 5 months of feeding, colonies were moved to individual experimental aquaria within the Red Sea Simulator (Bellworthy & Fine, 2018) with the same abiotic conditions as the troughs. Water flow into aquaria was maintained and the overflow drain covered with a 200 µm mesh filter to retain released planulae. Planulae collection took place over six consecutive nights. Planulae were collected in May 2016 using a disposable Pasteur pipette and counted within 1–2 hr of release (ca. 22:00 to 01:00 hr). Planulae for further analyses were pooled from all parents within each treatment into ice cold seawater and briefly rinsed with double distilled water (DDW), snap‐frozen in liquid nitrogen, and stored in batches of 10 individuals in preweighed Eppendorfs at −80°C. Planulae (*n* = 120 per feeding regime) were also collected into ambient seawater to be sorted for settlement experiments (see below). The maximal photochemical efficiency of individual newly released planulae (*n* = 10 individuals per feeding regime) was determined using a chlorophyll fluorescence imaging system (Microscopy Imaging‐PAM; Walz GmbH). Planulae were first dark adapted for 20 min prior to measuring the maximum photochemical yield with a single saturating light pulse (PAM settings: measuring light intensity, 10; measuring frequency, 8; actinic light intensity, 12; width, 0; gain, 12; dampening, 5). For all analyses, planulae from all colonies within a single feeding regime were combined. Upon termination of planulae collection, a 5 cm fragment of each parent colony was snap‐frozen in liquid nitrogen and stored at −80°C for subsequent analyses.

### 2.2 Parental investment and maternal condition

Coral tissue was completely removed from maternal colony fragments using an air brush with filtered seawater (0.2 μm FSW). The skeleton was retained for surface area determination using the paraffin wax dip method of Stimson and Kinzie (Stimson & Kinzie, 1991) and calibrated against a standard linear curve for objects of a known surface area and corresponding wax weights. The tissue slurry was electrically homogenized before centrifugation (at 3,000 *g* for 5 min). A 100 μl sample of the supernatant was taken for host protein analysis using the Bradford protocol (Bradford, 1976). Triplicate samples of 4 μl were mixed with 200 μl dye reagent (Coomassie Brilliant Blue G‐250) in a 96‐well plate and incubated for 30 min in the dark at room temperature before the optical density was read using a BioTek Synergy H1 fluorescence reader at 595 mm (BioTek Instruments Inc.). The protein analyses were calibrated with a standard curve obtained with bovine serum albumin and normalized to coral skeleton surface area. The remaining supernatant was discarded and the pellet suspended in 40 ml 0.2 μm FSW between each subsequent washing and centrifugation (2 × 5 min at 3,000 *g* and 1 × 5 min at 5,000 *g*). The final pellet was suspended in 2 ml 0.2 μm FSW and homogenized before subsamples were taken for Chl *a* (1 ml) and symbiont cell (100 μl) counts. Algal symbiont abundance in planulae samples was determined using a haemocytometer (Improved Neubauer) and in adults flow cytometry was used (Attune NxT autosampler; Life Technologies). Samples for Chl *a* analysis were centrifuged (10,500 *g*, 30 s, 4°C) and suspended in 1 ml 90% (v:v) acetone: DDW. Chl *a* was extracted for 20 hr at 4°C and measured with a spectrophotometer (Ultrospec 2100 Pro, Biotek, Lumitron) according to Jeffrey and Humphrey (Jeffrey & Humphrey, 1975). Chl *a* concentrations were normalized to total protein content or surface area of each fragment.

Planulae were analyzed using the same methods as above with the exclusion of the requirement to remove tissue from the skeleton and the following slight modifications. Triplicates of 20 planulae per feeding treatment were homogenized in 1 ml FSW from which total protein samples were taken. The final pellet was resuspended in 1 ml FSW, a 40 µl cell count sample was removed before adding acetone for Chl *a* extraction and analysis as above.

### 2.3 Influence of temperature on planulae settlement and mortality

To assess whether parental feeding regime affects time to settlement and mortality rates at different temperatures, depending on the availability of planulae, 5–15 individuals from either fed or unfed parental colonies were placed into a settlement chamber (50 ml Falcon tube with mesh ends) and held in experimental tanks. While chamber confinement does not exactly simulate of the natural environment, it enables multiple replicates within the experimental set up and creates comparability to previous studies, for example, (Fabricius, Noonan, Abrego, Harrington, & De'ath, 2017; Foster, Falter, McCulloch, & Clode, 2016; Foster, Gilmour, Chua, Falter, & Mcculloch, 2015; Graham, Baird, & Connolly, 2008). Each aquarium contained triplicate settlement chambers of either planulae from fed or unfed parents (total *n* chambers = 24, 12 per feeding group). A temperature gradient was chosen to emulate an extension of the full annual thermal range in the GoA. Temperatures of −3, 0, +3, and +6°C relative to ambient (24–25°C) were set in the experimental tanks of the Red Sea Simulator controlled on an individual aquaria basis by titanium heat exchangers (details within Bellworthy & Fine, 2018). These temperatures represented the sea surface temperature in Eilat during February ca. 22°C (−3°C), July ca. 27°C (3°C), and +3°C of the summer maxima at ca. 31°C (6°C). Chambers contained pieces of ceramic tile that had been preconditioned in the flow through troughs for 5 weeks prior to the initiation of settlement trials.

Percentage settlement and mortality was determined every 24 hr in all temperature treatments until all planulae had settled, died, or monitoring ended after 5 days. Settlement was defined as complete metamorphosis to a proto‐polyp and adhesion to the substrate. Since planulae degrade rapidly after death (Yakovleva et al., 2009), if planulae were absent, they were assumed to be dead (Cumbo, Edmunds, Wall, & Fan, 2013).

### 2.4 Lipid extraction, separation, and derivatization of fatty acids

Planulae from parental colonies were collected during the experimental phase and stored for lipid analyses. Coral tissue and symbionts were not obtained from experimental colonies but were taken from colonies of the same species and located in the same flow through troughs where the parents were held. Planulae samples (0.32–3.05 mg dry weight, DW), preheated GFF glass fiber filters (45 mm in diameter and <0.7 μm in pore size) containing coral host or symbiont tissue (156–304 mg DW), 5 ml of coral food mixture (*Artemia* nauplii at a concentration of ca. 500 individuals/ml), or particulate organic matter from the flow through troughs (1,500 ml filtrate) were frozen at –20°C for 1 day, freeze‐dried for 2 days and stored at –20°C before lipid extraction. The lipid analyses were performed at the Institute for Earth Science Dynamics, University of Lausanne, Switzerland, using procedures adapted from Spangenberg, Ferrer, Jacomet, Bleicher, and Schibler (2014) and Spangenberg, Jacomet, and Schibler (2006). Organic solvents suitable for chromatography (>99%) (Merck/VWR Chemical) were glass‐distilled shortly before use. Purified water (MQW) was obtained with a Direct‐Q UV 3 Millipore^®^ System (Merck). All the glassware used for sample handling was thoroughly washed, rinsed with deionized and purified water, and heated at 480°C for at least 8 hr before use.

The samples were spiked with an aliquot of an internal standard solution containing a defined amount of deuterated carboxylic acids (deuterated lauric acid, D_23_C_12:0_ and deuterated arachidic acid, D_39_C_20:0_, Cambridge Isotopes Laboratories) in dichloromethane and extracted using sonication in solvents of decreasing polarity (1 × 10 min in 6 ml MeOH, 2 × 10 min in 6 ml MeOH/CH_2_Cl_2_, 1:1, v/v, 1 × 10 min in 6 ml CH_2_Cl_2_). The extracts were combined and the solvent removed via gentle evaporation under clean N_2_ flow. The fatty acids (FA, carboxylic acids) were obtained by hydrolysis with 1 M KOH (95% EtOH) at room temperature for 16 hr. The nonsaponifiable lipids were separated with hexane aliquots (1 × 4 ml, 2 × 2 ml) after addition of 3 ml MQW. The hexane extracts were combined, washed with MQW, and dried with anhydrous Na_2_SO_4_ and solvent evaporated to dryness under gentle N_2_ flow. The fraction containing the acid lipids was acidified (to pH 2) with 6 N HCl, and the acid lipids extracted with hexane (1 × 4 ml, 2 × 2 ml) and methylated (with 14% BF_3_/MeOH) to provide fatty acid methyl esters (FAMEs). The FAMEs were stored at –20°C until analysis.

### 2.5 Chemical characterization and quantification of fatty acids

Chemical characterization and quantification of the FAMEs was performed by gas chromatography/mass spectrometry (GC/MS). The GC/MS instrument consisted of an Agilent 6890 gas chromatograph connected to an Agilent 5973 mass selective detector operating at 70 eV (source at 230°C and quadrupole 150°C) in the electron ionization mode (emission current 1 mA, multiple ion detection over m/z 20 to 550), and helium as carrier gas. A Zebron™ ZB‐FAME (Phenomenex Helvetica GmbH) was used for FAMEs separation with a fused‐silica column (30 m × 0.25 mm i.d.) and coated with a 0.2 μm high cyanopropyl stationary phase. This ZB‐FAME column is dedicated to an improved separation of cis/trans FAME and PUFA isomers. Samples were injected at 240°C with a split ratio of 50:1. After a first period of 2 min at 60°C, the column was heated to 140°C at 10°C/min, then to 190° at 3°C/min (held for 4 min), and finally to 260°C at 30°C/min (held for 6 min). The FAMEs were next analyzed with another fused‐silica column and GC temperature program to confirm the detection of long chain FAs (i.e., PUFAs). A HP‐ULTRA 2 (50 m × 0.32 mm i.d.) coated with 0.17 µm cross‐linked 5% diphenyl/95% dimethyl polysiloxane stationary phase (Agilent Technologies). Samples were injected splitless at 320°C. After an initial period of 2 min at 100°C, the column was heated to 310°C (held 20 min) at 4°C/min. Compound identification was based on comparison with standard mass spectra in the NIST14 Mass Spectral Library (National Institute of Standards and Technology), GC retention time (RT), and MS fragmentation patterns. Assignment of the polyunsaturated FA (PUFAs) isomers was done by comparison of the RTs and MS spectra of the Supelco 37 Component FAME mix (Sigma‐Aldrich), containing FAME's in the C_4_–C_24_ range. The concentrations of the FAs were determined from the peak area ratios of unknown and internal standards (deuterated lauric acid and deuterated arachidic acid) of known concentrations.

The absence of any measurable extracted lipid from the blank samples run for every six samples through the whole analytical procedure indicates that no detectable laboratory contamination was introduced in the samples.

### 2.6 Statistical analyses

Significance of the physiological differences between parent corals and in planulae from fed and unfed parents (“Feeding” factor, Table 1) were tested using one‐way ANOVA. The effect of parental feeding and incubation temperature (“Temperature” factor) and their interaction upon total mortality of planulae were tested using a two‐way ANOVA (Table 1). Since the temporal dynamics of planulae settlement can influence predation risk in the planktonic phase, dispersal, and reef connectivity, planulae time to settlement was assessed using Gehan–Breslow Survival Curves. Holm–Sidak pair wise comparisons were used to distinguish the significant groups following two‐way ANOVA and survival tests. All data were tested and passed the parametric assumptions of equal variance and normality before ANOVA's were conducted with one exception; after failing normality, Kruskal–Wallis ANOVA on ranks were used to compare total concentration of FA groups between host tissue and planulae. Results were considered significant with a *p* value of <.05. Data analyses were performed using SigmaPlot v12.5 software (Systat software Inc.). All values are reported as mean ± standard error of mean (*SEM*) unless otherwise stated. Planulae obtained from unfed parents are denoted as “*U* planulae” and those from fed parents as “*F* planulae.”

**Table 1:** Summary of statistical tests

	Test	Test factor	SS	*df*	*F*	*p*
Parents						
Chlorophyll *a*/cell	One‐way ANOVA	Feeding	819.788	11	3.442	.093
Cells/cm^2^	One‐way ANOVA	Feeding	4.50E+12	11	4.087	.071
Chlorophyll *a*/cm^2^	One‐way ANOVA	Feeding	194.295	11	0.009	.982
Host protein/cm^2^	One‐way ANOVA	Feeding	0.396	11	9.306	.012*
Cells/protein	One‐way ANOVA	Feeding	1.97E+13	11	5.784	.037*
Chlorophyll *a*/protein	One‐way ANOVA	Feeding	25,576.092	11	5.076	.048*
Planulae						
Chlorophyll *a*/cell	One‐way ANOVA	Feeding	11.576	5	8.918	.040*
Cells/planula	One‐way ANOVA	Feeding	6.42E+06	5	0.714	.446
Chlorophyll *a*/planula	One‐way ANOVA	Feeding	0.017	5	16.855	.015*
Protein/planula	One‐way ANOVA	Feeding	249.134	5	4.129	.112
Cells/protein	One‐way ANOVA	Feeding	2.86E+07	5	1.288	.320
Chlorophyll *a*/protein	One‐way ANOVA	Feeding	1,587.245	5	0.996	.375
*F*_v_/*F*_m_	One‐way ANOVA	Feeding	0.146	19	10.397	.005**
Fatty acids	One‐way ANOVA	Feeding	24,581.016	5	24.293	.008**
Mortality	Two‐way ANOVA	Feeding	412.181	1	1.939	.189
Mortality	Two‐way ANOVA	Temperature	1,577.784	3	2.474	.112
Mortality	Two‐way ANOVA	Feeding * Temperature	3,948.985	3	6.191	.009**

	Test	Test factor	Group	Events	Censored (%)	*df*	Test statistic	*p*
Settlement								
Time to settlement	Gehan–Breslow Survival	Temperature	Fed	180	20	3	30.922	<.001***
Time to settlement	Gehan–Breslow Survival	Temperature	Unfed	120	33	3	1.235	.745
Time to settlement	Gehan–Breslow Survival	Feeding	+6°C	75	29	1	5.944	.015*
Time to settlement	Gehan–Breslow Survival	Feeding	+3°C	75	13	1	0.153	.696
Time to settlement	Gehan–Breslow Survival	Feeding	Ambient	75	19	1	6.395	.011*
Time to settlement	Gehan–Breslow Survival	Feeding	−3°C	66	28	1	4.175	.041*

Note

Mature colonies of *Stylophora pistillata* were either supplied with Artemia feed (*Fed*) or not (*Unfed*) for 5 months (“Feeding” factor). The colonies and their resulting offspring (planulae) were tested for the physiological parameters listed. Planulae were also exposed to different temperatures (ambient, 6 and 3°C above ambient, and 3°C below ambient) to assess the effect of temperature upon mortality and time to settlement.

* *p* = <.05,

** *p* = <.01,

*** *p* = <.001

## 3 RESULTS

### 3.1 Maternal characteristics

**Figure 1:**
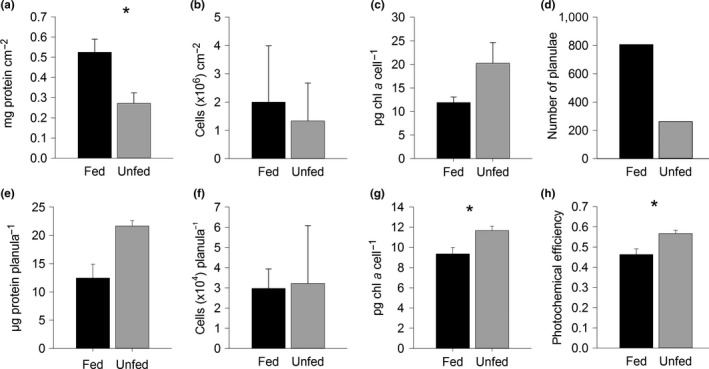
Parent (a–d) and planulae (e–h) physiology from fed (black) and unfed (gray) *Stylophora pistillata*: (a) parent host protein concentration per surface area, (b) parent symbiont cells per surface area, (c) parent chlorophyll *a* concentration per symbiont cell (parents fed *n* = 6, unfed *n* = 6), (d) total number of planulae released, (e) protein concentration per planula, (f) symbiont cells per planula, (g) chlorophyll *a* concentration per symbiont cell in planulae (*n* = triplicate of 20 pooled planulae per treatment), and (h) photosynthetic yield of planulae (*n* = 10 per treatment. Asterisks indicate significant differences between fed and unfed colonies (one‐way ANOVA). Data are mean ± *SEM*

Chl *a* concentration per surface area between fed and unfed parent colonies was not different, but host protein (Figure 1a) almost doubled in fed colonies (0.524 ± 0.025 mg protein/cm^2^) compared to unfed colonies (0.272 ± 0.016 mg protein/cm^2^; Figure 1a). Lower areal protein concentration but sustained symbiont cell density/cm^2^ (Figure 1b) resulted in significantly higher cell density/host protein content in the unfed colonies (Table 1). Unfed colonies had a nonsignificant increase in Chl *a* concentration per symbiont cell (20.25 ± 4.36 pg Chl *a*/cell) versus fed colonies (Figure 1c; 11.89 ± 1.16 pg Chl *a*/cell, *p* = .093). Reproductive output was more than three times lower from unfed corals (262 planulae) than fed corals (807 planulae) from all colonies combined over the 6‐day sampling period (Figure 1d).

### 3.2 Planulae characteristics

Although symbiont cell counts did not differ between feeding regimes (Figure 1f; fed: 29,683 ± 1,668; unfed: 32,229 ± 4,946 cells/planula), Chl *a* concentration was significantly greater in *U* planulae (18.67 ± 1.07 ng Chl *a*/planula) compared to *F* planulae (13.82 ± 0.51 ng Chl *a*/planula, *p* = .015; Table 1). *U* planulae also had significantly higher Chl *a* content per symbiont cell than *F* planulae (11.66 ± 0.44 and 9.35 ± 0.64 pg Chl *a*/cell respectively, *p* = .041; Figure 1g). Photosynthetic efficiency was significantly higher in *U* planulae (*U*: 0.57 ± 0.02; *F*: 0.46 ± 0.03, Figure 1h). The mean total protein per planula was 43% lower in *F* planulae; however, large sample variability made this difference statistically insignificant (Figure 1e, Table 1).

### 3.3 Settlement and mortality rates

**Figure 2:**
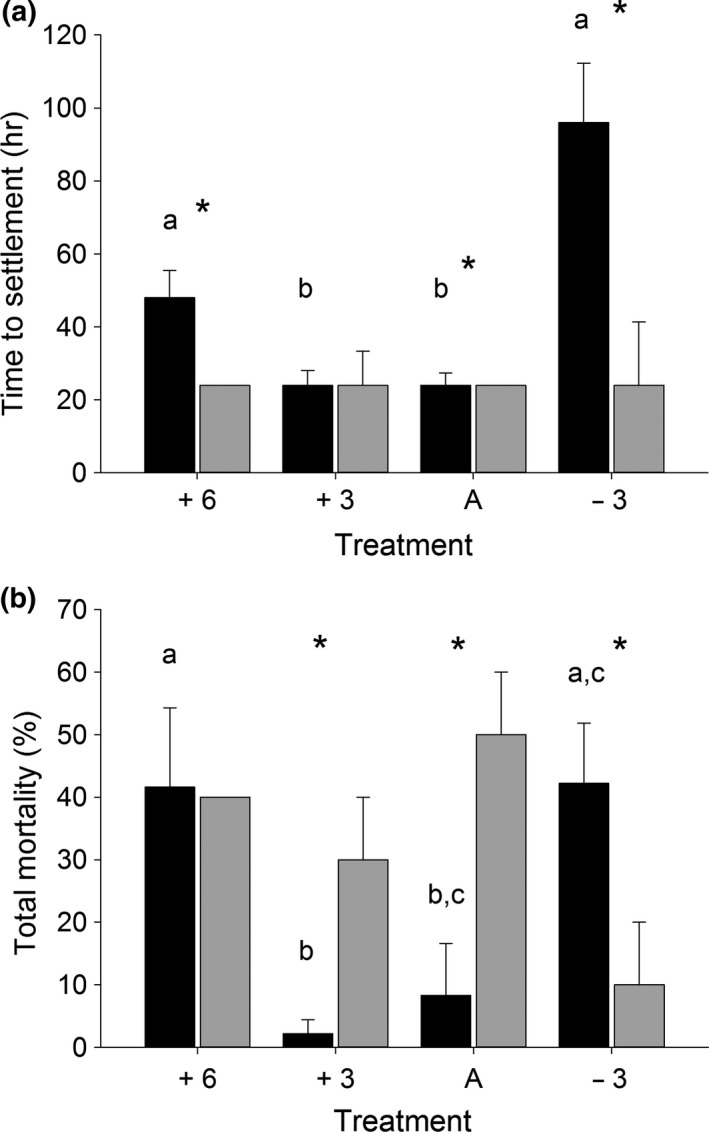
Planulae median time to settlement (a) and total mortality (b) from fed (black, *n* = 180) and unfed (gray, *n* = 120) parent colonies. Asterisks indicate significant differences between feeding type and different letters indicate differences within a feeding type between temperatures (time to settlement: Gehan–Breslow survival curve; total mortality: two‐way ANOVA, *p* = <.05). +6 (6°C above ambient, ca. 30°C), +3 (3°C above ambient, ca. 27°C), A (ambient seawater temperature, ca. 24°C), and −3 (3°C below ambient, ca. 21°C). Data are median ± *SEM* in (a) and mean ± *SEM* in (b). There are no error bars present in the cases where all planulae have settled within the first 24 hr observation point

Parental diet significantly influenced median time to settlement as *F* planulae took significantly longer to settle in all cases except at +3°C above ambient where the difference between feeding groups was not significant (asterisks Figure 2a). Despite the same median value between feeding groups at ambient temperature, a significant difference is recorded as 100% of *U* planulae settled within 24 hr, whereas in *F* planulae 77% settled in 24 hr and took up to 144 hr to settle. In *F* planulae, time to settlement was significantly longer at the thermal extremes of +6 and −3°C compared to ambient and +3°C (Figure 2a). Temperature did not significantly affect time to settlement in *U* planulae, where median time to settlement was always <24 hr regardless of temperature (Figure 2a).

The effect of temperature upon planulae mortality was dependent upon parental diet (two‐way ANOVA, *p* = .009, Figure 2b, Table 1). Total mean mortality for *U* planulae within treatments ranged between 10% and 50% but with no significant temperature differences. Within *F* planulae, temperature significantly affected mortality (Table 1, two‐way ANOVA, *p* = <.001). Mortality was significantly higher at +6°C (42%) and −3°C (42%) but lower in the +3°C (2.2%) and ambient treatments (8.3%). Mortality was not significantly different between *F* and *U* planulae in the +6°C treatment, but was significantly higher in *U* planulae at +3°C and ambient temperature and, conversely, significantly higher in *F* planulae in the −3°C treatment.

### 3.4 Fatty acid distribution and abundances

Figure S1 depicts the total ion chromatograms of the saponifiable lipid (including free fatty acids, glycerides, phospholipids, sphingolipids, and wax esters) and unsaponifiable lipid faction (i.e., alcohols, sterols). For discussion the FA were grouped by their degree of unsaturation, which may be related to their origin and functionality. All coral samples contained similar FAs in the C_14_ to C_24_ range (Tables S1 and S2). In all samples, the main saturated fatty acids (SAFAs) were straight chains with >12 even carbon number, including 14:0 (myristic acid), 16:0 (palmitic), 18:0 (stearic), 20:0 (arachidic), 22:0 (behenic), and trace amount of 24:0 (lignoceric); the most abundant was 16:0. The monounsaturated FAs (MUFAs) were 16:1, 18:1, 20:1, 22:1, and 24:1 isomers, clearly maximizing at oleic acid (18:1*n*‐9). The quantified polyunsaturated FAs (PUFAs, with ≥2 double bonds) were 18:2, 20:2, 18:3 isomers, 20:3*n*‐6, 20:4*n*‐6, and the *n*‐3 PUFAs stearidonic acid (SDA, 18:4*n*‐3), eicosapentaenoic acid (EPA, 20:5*n*‐3), and docosahexaenoic acid (DHA, 22:6*n*‐3).

**Figure 3:**
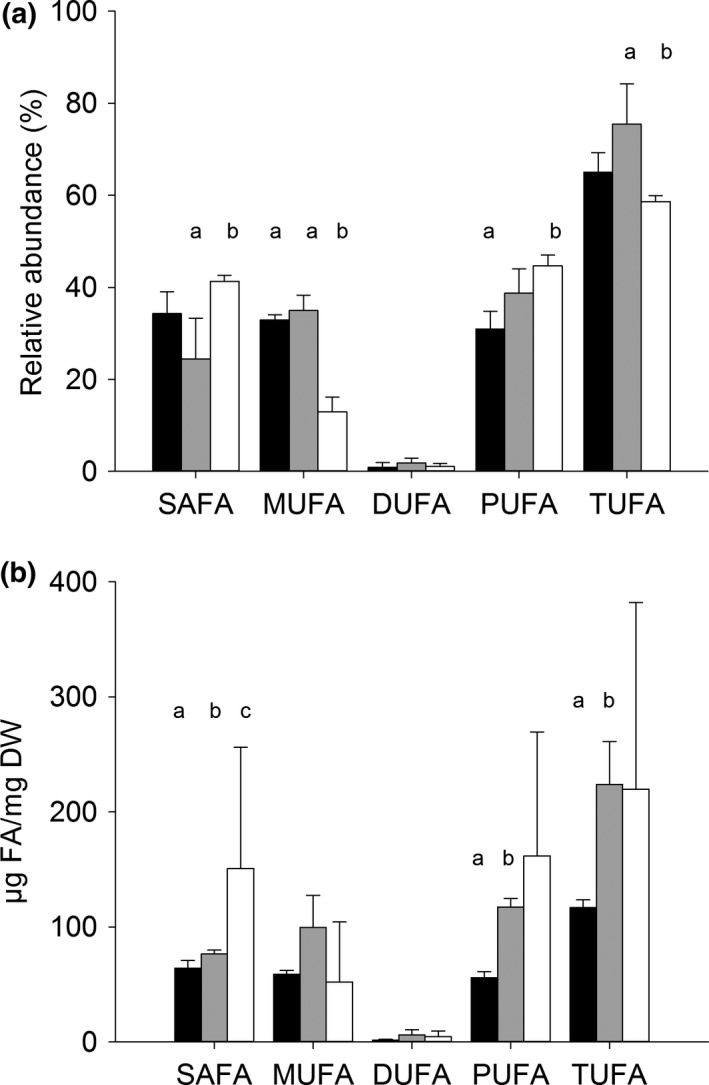
Percentage relative abundance (a) and concentration (b) of fatty acid groups in planulae from fed (black, *n* = 3) and unfed (gray, *n* = 3) parent colonies and within coral host tissue (white, *n* = 5). SAFA (saturated), MUFA (monounsaturated), DUFA (diunsaturated), PUFA (polyunsaturated), and TUFA (total unsaturated). Different letters indicate significant differences (ANOVA, *p* = <.05) between samples within a fatty acid group. Data are mean ± *SD*

Adult coral host tissue contained a lower percentage of MUFAs (12.98 ± 1.41%) compared to both *F* planulae (32.91 ± 0.65%) and *U* planulae (34.64 ± 1.77%) (Holm–Sidak post hoc: host vs. *F*: *t* = 9.88, *p* = <.001; host vs. *U*: *t* = 10.73, *p* = <.001; Figure 3a, Table 2) Adult corals contained relatively more SAFAs (41.33 ± 0.57%) compared to *U* planulae (24.40 ± 5.16%, Holm–Sidak post hoc: *t* = 4.503, *p* = .006) and a higher percentage of PUFAs (44.63 ± 1.08%) than *F* planulae (31.84 ± 2.26%, Holm–Sidak post hoc: *t* = 4.315, *p* = .008). Only within SAFA was the total concentration significantly higher in host tissue per mass dry weight compared to *U* and *F* planulae (Kruskal–Wallis ANOVA on Ranks, *H* = 5.0, *df* = 1, *p* = .036; Figure 3b). Specifically, the most abundant FAs in adult coral tissue were 16:0 (18.2%), 18:0 (17.4%), 20:4*n*‐6 (16.3%), 20:5*n*‐3 (6.2%), 22:6 cis isomers (8.6%), and 22:6*n*‐3 (6.9%). *F* and *U* planulae combined contained 19.7% 16:0 similar to adult colonies. Yet, in comparison to adult tissues, planulae were enriched in 18:1*n*‐9 (18.9%) and 22:6*n*‐3 (23.4%), which was the most abundant FA in all samples. Planulae contained comparatively lower abundances of the other FAs dominant in adults.

**Table 2:** Mean relative abundance (top) and concentration (bottom) of fatty acid groups in planulae from unfed (*n* = 3) and fed (*n* = 3) parent colonies, coral host tissue (*n* = 5), symbiont tissue (*n* = 5), Artemia coral feed (*n* = 3), and trough water (seawater [*n* = 1]) and ratios between them

Relative abundance (%)	Unfed planulae		Fed planulae		One‐Way ANOVA			Host tissue		Symbiont tissue		Artemia		Seawater
	Mean	*SD*	Mean	*SD*	SS	*F*	*p*	Mean	*SD*	Mean	*SD*	Mean	*SD*	Mean
SAFA	24.39	8.93	34.27	4.81	352.1	2.854	.167	41.33	1.28	28.64	5.57	27.63	3.06	86.60
MUFA	34.97	3.34	32.91	1.12	25.9	0.834	.370	12.98	3.15	9.05	2.15	22.34	0.51	9.53
DUFA	1.78	1.14	0.88	1.08	6.148	1.007	.372	1.07	0.63	3.62	1.37	4.18	0.28	0.00
PUFA	38.71	5.34	30.96	3.85	221.1	4.140	.111	44.63	2.40	41.88	3.08	45.00	2.51	2.83
TUFA	75.47	8.75	65.01	4.27	353.6	3.461	.142	58.67	1.28	54.70	5.57	71.95	3.02	13.40
SAFA: MUFA	0.72	0.33	1.04	0.16	0.425	2.318	.201	3.35	0.85	2.80	1.30	1.24	0.16	9.08
MUFA: DUFA	10.50	7.97	36.09	21.92	2,069.7	3.610	.130	9.55	1.29	2.22	0.74	5.36	0.33	
DUFA: PUFA	0.04	0.03	0.03	0.03	0.004	0.404	.600	0.02	0.01	0.07	0.03	0.09	0.004	
MUFA: PUFA	0.91	0.10	1.07	0.14	0.124	4.636	.172	0.29	0.09	0.18	0.04	0.50	0.02	3.37
SAFA: TUFA	0.34	0.16	0.53	0.11	0.137	2.892	.164	0.71	0.04	0.44	0.13	0.39	0.06	6.46
PUFA: SAFA	1.78	0.83	0.92	0.22	3.038	3.171	.159	1.08	0.05	1.25	0.32	1.65	0.27	0.03
SAFA: PUFA	0.66	0.32	1.13	0.31	0.732	3.409	.139	0.93	0.04	0.58	0.15	0.62	0.10	30.62

Total concentration	Unfed planulae		Fed planulae		One‐way ANOVA			Host tissue		Symbiont tissue		Artemia		Seawater
	mg FA/g DW		mg FA/g DW					µg FA/g DW		µg FA/g DW		µg FA/ml		µg FA/L
	Mean	*SD*	Mean	*SD*	SS	*F*	*p*	Mean	*SD*	Mean	*SD*	Mean	*SD*	Mean
SAFA	76.40	3.51	64.19	6.51	333.3	8.177	**.046**	150.86	105.15	115.32	102.80	35.64	11.50	0.79
MUFA	99.61	27.81	58.88	3.24	3,816.3	6.446	.064	52.25	52.02	32.70	18.83	29.25	10.17	0.09
DUFA	6.12	4.57	1.48	0.67	80.54	2.666	.178	4.56	4.92	11.68	4.79	5.55	2.22	0.00
PUFA	117.38	7.23	55.80	5.47	6,892.1	83.37	**<.001**	161.59	107.91	160.74	109.66	59.32	21.94	0.02
TUFA	223.70	37.46	116.67	6.91	19,895.5	23.42	**.009**	219.61	162.34	206.01	132.33	94.81	34.58	0.12
SAFA: MUFA	0.81	0.23	1.09	0.86	0.238	3.674	.127	3.35	0.85	3.36	1.30	1.24	0.16	9.08
MUFA: DUFA	31.19	30.48	44.94	42.09	3,240.8	0.383	.569	2.40	2.06	2.67	0.74	5.36	0.33	
DUFA: PUFA	0.05	0.04	0.03	0.01	0.006	0.688	.453	0.02	0.01	0.09	0.03	0.09	0.004	
MUFA: PUFA	0.84	0.21	1.06	0.79	0.164	4.550	.194	0.29	0.09	0.22	0.04	0.50	0.02	5.42
SAFA: TUFA	0.35	0.05	0.55	0.45	0.079	15.33	**.017**	0.70	0.04	0.53	0.13	0.39	0.06	6.46
PUFA: SAFA	1.54	0.05	0.88	0.52	0.830	42.17	**.004**	1.08	0.05	1.50	0.32	1.65	0.27	0.02
SAFA: PUFA	0.65	0.02	1.16	0.96	0.495	15.82	**.016**	0.93	0.04	0.69	0.15	0.62	0.10	49.25

Note

Ratios are averages from raw data not mean values. *p* value represents results from a one‐way ANOVA between fed and unfed planulae samples (*df* = 5). Significant results are marked bold.

Abbreviations: DUFA, diunsaturated; MUFA, monounsaturated; PUFA, polyunsaturated; SAFA, saturated; TUFA, total unsaturated.

**Figure 4:**
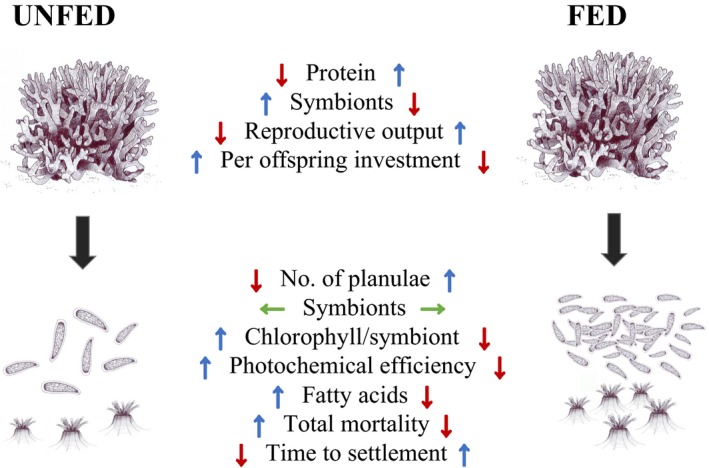
Experimental results summary highlighting the differences found between corals fed twice per week with *Artemia* nauplii (fed) and unfed (supplied filtered natural seawater [500 µM]). For each parameter, red negative arrows indicate a significant decrease, blue positive arrows indicate a significant increase, and green horizontal arrows indicate no significant difference between parental diets. Parameters were tested with one‐way ANOVA as a function of diet, except total mortality (two‐way ANOVA, diet and incubation temperature) and time to settlement (Gehan–Breslow survival curve). The latter two tests have multiple outputs; the arrows here indicate overall trend

Although the relative abundance of FAs in planulae was not significantly impacted by parental diet the absolute concentration of SAFA (particularly 16:0), PUFAs (particularly 22:6*n*‐3), and TUFA were significantly higher in *U* planulae than *F* planulae (*p* = <.05, Table 2, Figure 3b). Correspondingly, average total fatty acid concentration was significantly higher (*p* = <.008) in *U* planulae (299.5 ± 23.7 µg FA/mg DW) than *F* planulae (180.8 ± 4.2 µg FA/mg DW). These concentrations correspond to 26.7 ± 5.1 µg FA/*U* planula and 10.4 ± 3.1 µg FA/*F* planula. All results are summarized in Figure 4.

## 4 DISCUSSION

### 4.1 Effect of diet in parents and planulae

Mature colonies fed a supplementary zooplankton diet (*Artemia* sp. nauplii) significantly increased protein concentration likely as a direct result of the additional intake of nitrogen, phosphorous, and amino acids (Houlbrèque & Ferrier‐Pagès, 2009). Unfed colonies on the other hand, harbored denser symbiont populations (cells mg/total protein, *p* = .037; cells/surface area^2^, *p* = .071) and showed tendency toward higher Chl *a* concentration (Chl *a*/host protein, *p* = .048; Chl *a*/cell, *p* = .093). Even though significant physiological changes were observed, the feeding treatment is likely a conservative representation of food availability on the reef, where corals have access to a quasi‐continuous availability of diverse zooplankton. In previous studies, feeding with similar diets to that used here, has been reported to increase (Treignier, Grover, Ferrier‐Pagés, & Tolosa, 2008), decrease (Al‐Moghrabi, Allemand, Couret, & Jaubert, 1995), and not change (Krueger et al., 2018) symbiont densities and/or Chl *a* concentration and the outcome likely depends on other environmental factors such as light level. Increased Chl *a* concentration found in this study may be an acclimation to low food conditions and low light in an attempt to increase light capture and photosynthetic efficiency. Indeed, symbionts in planulae from unfed parents had significantly higher photochemical efficiency compared to symbionts in *F* planulae. Likewise, *U* planulae also had significantly higher Chl *a* concentration per planula and per symbiont cell. The similar trend in per cell Chl *a* concentration between parents and planulae is likely a result of the direct vertical transmission of symbionts in this species.

Interestingly, diet did not significantly change the relative abundance of FA groups. This indicates that the component make up of planulae is rather consistent and not significantly influenced by parental diet. Parental diet did however significantly alter total fatty acid concentration per planulae; *U* planulae had significantly higher total lipid concentration of 26.7 ± 5.1 µg FA/planula (30.0 ± 2.4% DW as FA) compared to 10.4 ± 3.1 µg FA/planula for *F* planulae (18.1 ± 0.4% DW as FA). These values fall close to the range of previously published concentrations of 12.5–91.5 µg total lipid/planula from *P. damicornis* (Harii et al., 2010; Rivest, Chen, Fan, Li, & Hofmann, 2017; Rivest & Hofmann, 2015), which produce relatively large brooded planulae and also on the lower side of the percentage of DW range (27.4%–35.5%) reported from four species of Australian *Acropora* (Conlan, Humphrey, Severati, & Francis, 2017). Lipids are typically associated with metabolic energy, growth, membrane structure, reproduction, and immune functions. The current study indicates that *S. pistillata* planulae have lower fatty acid (lipid) concentrations compared to other species and this energy reserve may be adequate for their short presettlement period (see Section 4.2). Specifically, unfed planulae had higher SAFA, PUFA, and TUFA concentrations. Coral symbionts and their photosynthates contain high proportions of PUFA (Papina, Meziane, & van Woesik, 2003; Teece, Estes, Gelsleichter, & Lirman, 2011; Treignier et al., 2008), which include coral essential FA's, important to maintaining appropriate membrane fluidity in a variable environment and also as available energy (Treignier et al., 2008). Therefore, it can be suggested that high PUFA concentrations in *U* planulae are indicators of photosynthetically derived energy and are consistent with the observed higher symbiont densities in the unfed parent colonies and their offspring. There is potential for future research to investigate the suitability of using PUFA abundance as a proxy for quantifying the relative contribution of heterotrophy to autotrophy to a coral's energy intake.

Palmitic acid (16:0), essential to membrane phospholipids, was the most abundant fatty acid in adult host, symbiont, and *F* planulae and the second most abundant in *Artemia* after 18:3*n*‐3. This is in agreement with previous studies, for example, (Al‐Moghrabi et al., 1995) and can therefore be considered a dominant, preserved FA. In *U* planulae, however, while they still contained an average of 16.3 ± 3.9% palmitic acid, the most abundant FA was DHA (22:6*n*‐3) with 28.9 ± 3.0%. DHA was also the second most abundant FA in *F* planulae (17.9 ± 1.6%). These relative abundances are considerably higher than in adult host tissue in this (6.9 ± 1.0%) and previous studies (e.g., Al‐Moghrabi et al., 1995; Bachok, Mfilinge, & Tsuchiya, 2006; Papina et al., 2003). DHA is crucial for growth, survival, and early development in marine larvae (Sargent et al., 1999) which may relate to its preferential concentration in planulae. Currently, there is only one other study which details the FA profile in coral planulae; using four *Acropora* species, Conlan et al. (2017) report highly contrasting trends in FA relative abundance compared to the present study and also report substantial shifts in FA distribution postsettlement. For example, DHA in *Acropora* planulae approximated 2% and EPA (20:5*n*‐3) contributed ca. 9% compared to ca. 1% in *S. pistillata* from this study. With no other comparative data, it is difficult to ascertain the reasons for these differences. Additional similar studies on multiple coral species from varying parental environments may help elucidate the influencing mechanisms.

The number of planulae released from fed colonies was three times higher compared to unfed colonies. However, as detailed above, individual *F* planulae had significantly lower total fatty acid concentrations; using these measures it is noted that, in contrast to the hypothesis, POI was unexpectedly higher in *U* compared to *F* planulae. This is different to bicolor damselfish (*Stegastes partitus*) where fed females did not significantly increase the number of offspring clutches but instead significantly increased lipid energy provisioning to each larva (Samhouri, 2017). At the other end of the feeding scale, damselfish (*Acanthochromis polycanthus*) on a minimal diet significantly reduced clutch size but not POI (egg size) compared to fish on an average diet (Donelson, Munday, McCormick, Pankhurst, & Pankhurst, 2010). These results indicate the diversity of ways in which reproductive output can be altered. While fish are evolutionarily distant from corals, this study presents the first results of the effect of parental diet upon POI in corals. It remains to be confirmed whether corals harbor a similar plasticity in offspring provisioning and how this is regulated in a diversity of environments. Other studies, not concerning parental diet, can offer an insight. POI was reported as stable in *Montipora capitata* eggs from Hawaii, as lipid reserves and antioxidant defense were equal between high light and shaded reefs despite adult plasticity (Padilla‐Gamiño et al., 2012). Contrastingly, variable POI was displayed in multiple coral species with higher lipid reserves allocated to larvae from healthier coral populations with higher benthic cover in Curacao (Hartmann, Marhaver, & Vermeij, 2018).

Increased POI, that is, planulae size, should only be selected for if it increases the fitness of the planulae within the prevailing environment (Monro, Sinclair‐Taylor, & Marshall, 2010). There are examples from planktonic arthropods where harsh environments such as those with low, unpredictable food availability have been shown to select for increases in POI while offspring number decreases (see Fox & Czesak, 2000, references therein). For example, low food levels caused *Daphnia hyalina* to produce smaller clutch sizes of larger eggs compared to a high food level treatment (Burns, 1995). There may be a few reasons for a similar trend to exist in corals. Firstly, increased lipid content in lecithotrophic larvae, as occurred here in unfed corals, may enable prolonged time to settlement (Isomura & Nishihira, 2001). Increased time to settlement gives rise to the potential for greater larval dispersal distances with increased chance to encounter preferable environmental conditions, that is, with higher food availability. However, in the field, increased pelagic larval duration comes with increased predation risk, complicating this environmentally driven trade‐off. Alternatively, increased POI may be used for rapid settlement, maintaining a larger size at metamorphosis (Marshall & Keough, 2007), linked to faster growth rates in colonial marine invertebrates (Marshall, Cook, & Emlet, 2006). Increased POI together with rapid settlement in *U* planulae from this study may result in increased juvenile size and faster growth, yet the effects of parental diet as a driver of postsettlement traits remains to be tested in corals. In contrast, low POI, as in the fed treatment herein, will likely shorten pelagic larval duration (PLD) and result in local retention of larvae within the high quality parent environment. Where environmental conditions are good, that is, higher food availability, there may be little need for high POI and producing greater numbers of smaller offspring instead enhances reproductive success and thereby parental fitness. Since mortality during the planktonic phase is high and stochastic, shorter PLD forced by lowered POI and increased offspring number are more likely to increase recruitment in good environments. This may drive a shift in Red Sea *S. pistillata* from r‐select (Loya, 1976) to more K‐select life history traits in low quality environments, which may be of potential consequence under climate change in this and other coral species. For instance, as an r‐strategist, *S. pistillata* is currently one of the most dominant species and among the first to occupy available substrate in the GoA; becoming a K‐strategist will likely reduce the abundance of this species on the reef and thereby alter the community structure.

### 4.2 Thermal response of planulae

Since mortality risk is typically highest in the pelagic phase, coral planulae are likely entrained to settle upon encountering a suitable environment. In the present study, as in many previous studies, planulae were placed into a small chamber relative to the size of the open, sea containing a settlement cue, and suitable substrate. Consequently, time to settlement within this experiment does not likely represent PLD in the field. This limitation should be considered before using experimentally determined settlement times in efforts to model dispersal and reef connectivity. However, the use of settlement chambers similar to other experimental set ups enables data comparison between coral species and locations.

Time to settlement and mortality were examined at four temperature deviations from ambient seawater (ca. 25°C). These temperatures represent an extension to which planulae from this population have previously been experimentally exposed (Bellworthy et al., 2019). Regardless of temperature, median time to settlement in *U* planulae was 24 hr postrelease, the first observation time point. Together with the observation of many planulae settling before collection, this implies that planulae of *S. pistillata* have full settlement competency upon release. This is in stark contrast to another brooding coral species, *Porites astreoides*, where after 24 hr at ambient temperatures (28°C) a median of 0% planulae settle (Edmunds, Gates, & Gleason, 2001). In other studies, elevated temperatures have been shown to increase development rates and increase mortality. Elevated temperatures of 33°C, 5°C above summer ambient temperature (28°C), caused a significant increase in mortality, metamorphosis rate, and decreased gross photosynthesis in planulae from *Porites astreoides* in Florida (Edmunds et al., 2001). In addition, temperatures of 32°C, 4°C above ambient, reduced time to settlement from 4 to 3 days in four broadcast spawning species from Okinawa and the Great Barrier Reef (Heyward & Negri, 2010) and increased mortality in *P. damicornis* from Taiwan (ambient 25°C, elevated temperature 29°C; Putnam, Mayfield, Fan, Chen, & Gates, 2013). Resistance of adult corals to above ambient temperatures has previously been identified in this population (Bellworthy & Fine, 2017; Fine et al., 2013; Krueger et al., 2017) and, in the absence of effects of temperature changes predicted within the next century upon planulae (see also Bellworthy et al., 2019), the current data begin to imply a similar resistance to ocean warming exists during early life history. It still however remains unknown whether postsettlement development may be a future bottleneck to the persistence of this climate change resilient population.

However, in *F* planulae, time to settlement was significantly longer at the thermal extremes both in comparison to *U* planulae and at +6 and −3°C (ca. 31 and 22°C respectively) compared to ambient and +3°C (ca. 25 and 28°C respectively) within the fed treatment. Significantly longer time to settlement at 22°C (ca. local winter minimum) in *F* planulae may indicate a slowed metabolism and an absence of the energetic reserves to increase development rate as *U* planulae did at this temperature. *F* planulae also had a longer time to settlement at 31°C (+6°C) compared to at 28°C (+3°C, maximum GoA summer temperature) and 25°C (ambient) temperatures. The reason for this is unclear; one hypothesis suggested here is that planulae with lower POI are driven to search for cooler waters that would reduce metabolic costs of metamorphosis and early life ontogeny and postpone settlement until conditions improve. It is possible that planulae are not as innately thermally plastic as adults in the GoA and therefore those with low POI do not as rapidly settle in an unfavorable environment and also suffer highest mortality rates at temperatures beyond the local annual range (Figure 2b). Since all internal brooding occurred at ambient temperatures, parents could not acclimate offspring to or anticipate that planulae may experience extreme settlement temperatures. It is possible however, with a warmer and more variable environment under future climate change scenarios, parental experience will produce planulae more capable of tolerating an unpredictable environment and that transgenerational acclimation and plasticity will rapidly alter planulae phenotype and improve fitness in warmer seas (e.g., Putnam & Gates, 2015).

The results demonstrate that the effect of temperature is dependent upon parental diet. This becomes an important factor when forecasting the effects of ocean warming on corals. Firstly, heterotrophic capacity is species dependant in part as a result of polyp size (Alamaru, Loya, Brokovich, Yam, & Shemesh, 2009) and ability to increase feeding rates (Grottoli et al., 2006). Feeding rates will also depend upon zooplankton availability in future oceans. Modeling efforts, together with some observational evidence (Richardson, 2008), suggest that markedly increased stratification in tropical waters caused by ocean warming will likely reduce nutrient concentrations in surface water, which will in turn lower phytoplankton productivity and therefore zooplankton abundance (Bopp, Aumont, Cadule, Alvain, & Gehlen, 2005). Therefore, it can by hypothesized that as ocean warming shifts zooplankton availability toward those of our unfed treatment, not only will increasing feeding rates potentially not be an option to offset autotrophic losses in bleaching, but that number of offspring may be reduced but with the POI to enable metamorphosis at increased seawater temperatures, and, to extrapolate our results further, result in increased local retention on reefs. Additional parental feeding experiments should be conducted using the expected future zooplankton community to ascertain effects of parental diet upon recruitment.

## 5 SUMMARY

This work is the first to investigate the carry over effect of parental feeding on reproductive output, per offspring investment, and offspring phenotype in a reef‐building coral. In addition, we report the effect of multiple temperatures upon time to settlement and planulae mortality in a coral species known to exhibit thermal tolerance during its adult life phase (Figure 2). We report high survival across all temperatures and that temperature did not significantly change time to settlement in the unfed group in contrast to previous reports for other coral species. This study affirms that carry over effects may be observed within a single gametogenesis cycle in corals, which increases the likelihood that corals may adapt to rapid global climate change. We also show that feeding enabled *S. pistillata* to more than triple reproductive output via increased planulae number but that unfed colonies provide higher POI predominantly in the form of fatty acids, protein, and symbiont density. These results synergistically raise the potential for the GoA to act as a thermal coral refuge and begin to elucidate the eco‐evolutionary reproductive choices employed by corals.

## CONFLICT OF INTEREST

None declared.

## AUTHOR CONTRIBUTIONS

JB conceived and carried out the experiment, physiological laboratory, data, and statistical analyses, manuscript and figure preparation; JES carried out lipid analysis by GC/MS, table preparation, and critically revised the written manuscript. MF helped design the experiment and critically revised the manuscript. All authors gave final approval for publication and agree to be held accountable for the work performed therein.

## Data Availability

Complete raw data are available online in the Dyrad Digital Repository (DOI: https://doi.org/10.5061/dryad.612r4t8).
