# Supplementary figures and images for: Feeding increases the number of offspring but decreases parental investment of Red Sea coral Stylophora pistillata
Source: Ecol Evol. 2019 Oct 2;9(21):12245–58. doi: 10.1002/ece3.5712 (PMC6854114; doi:10.1002/ece3.5712)

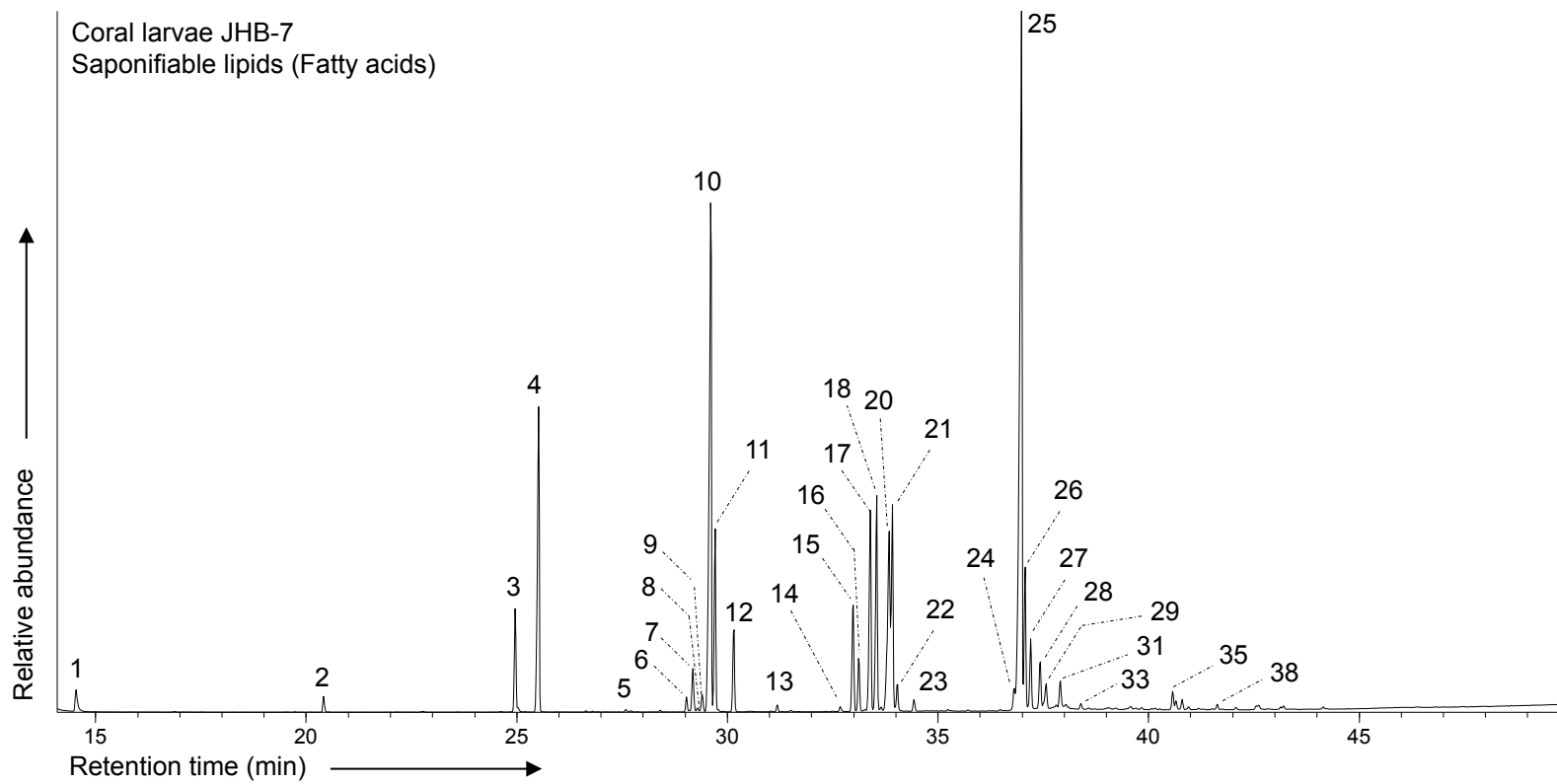

Supplement: Supplementary file 1 [file ECE3-9-12245-s001.pdf]
