# Supplementary material for: Feeding increases the number of offspring but decreases parental investment of Red Sea coral Stylophora pistillata
Source: Ecol Evol. 2019 Oct 2;9(21):12245–58. doi: 10.1002/ece3.5712 (PMC6854114; doi:10.1002/ece3.5712)
Supplement: Supplementary file 2 [file ECE3-9-12245-s002.pdf]

SI Table 1 Mean ± standard deviation relative abundance and concentration of individual fatty acids in planulae from unfed (*n* = 3) and fed (*n* = 3) parent colonies. Blank cells inidicate the compound was not found. The FAs are abbreviated as Cx:y, where óxô is the number of carbons and óyô the number of double bonds. The first double bond position is counted from the methyl end of the lipid alkyl chain (*n* or ɤ nomenclature).

Compound number	Compound name	Unfed Planulae				Fed Planulae			
		ug FA/ mg DW	st. dev.	Relative abundance %	st. dev.	ug FA/ mg DW	st. dev.	Relative abundance %	st. dev.
1	C14:0	3.01	0.11	1.07	1.38	2.72	0.03	1.56	0.67
2	C16:0	53.42	1.73	16.33	22.57	43.70	9.61	23.00	15.71
3	C16:1*n*-9					0.75	0.04	0.39	0.26
4	C16:1*n*-7	10.10	1.16	3.19	5.34	8.92	0.26	4.72	2.44
5	C18:0	10.87	2.26	3.34	3.25	9.54	1.18	5.08	1.79
6	C18:1*n*-9	58.39	20.92	20.70	6.54	29.72	3.70	17.14	10.45
7	C18:1*n*-4	11.88	0.92	4.08	4.33	9.48	0.38	5.01	2.70
8	C18:2*n*-9	1.87	0.74	0.62	0.19	0.36	0.52	0.22	0.52
9	C18:2*n*-6	1.50	1.42	0.36	0.32	1.09	0.19	0.64	0.48
10	C18:3*n*-6	1.96	0.73	0.58	0.35	1.27	0.15	0.74	0.51
11	C20:0	1.07	0.40	0.40	0.24	0.31	0.35	0.19	0.37
12	C18:4*n*-3	3.03	0.45	1.01	1.56	3.86	1.29	2.10	0.77
13	C20:1*n*-7 / *n*-3	12.75	5.22	4.65	0.78	6.93	1.10	3.98	1.37
14	C20:1*n*-11 / *n*-7	1.25	0.23	0.47	0.30	1.11	0.14	0.53	0.49
15	C20:2*n*-9	1.97	0.11	0.45	0.01	0.30	0.42	0.18	0.42
16	C20:2*n*-6	2.16	0.94	0.35	0.05	0.48	0.67	0.28	0.67
17	C20:3*n*-6	9.38	2.61	3.32	1.53	6.14	0.64	3.42	2.10
18	C20:4*n*-6	9.95	1.70	3.74	2.45	5.29	1.93	5.29	1.83
19	C22:0	6.81	0.38	2.88	2.88	6.97	1.69	3.91	0.57
20	C22:1*n*-9	1.10	0.99	0.33	0.29				
21	C20:5*n*-3	3.14	0.36	1.20	1.12	2.52	0.29	1.33	0.71
22	C24:0	1.22	0.33	0.37	0.36	1.05	0.62	0.59	0.25
23	C24:1*n*-9	4.15	0.90	1.55	0.90	3.09	1.48	1.72	0.65
24	C22:6*n*-3	89.92	4.91	28.86	33.75	32.20	3.22	17.93	9.17
	**Sum**	**299.51**	**40.96**	**100.00**		**180.85**	**7.32**	**100.00**	
