# Supplementary material for: Feeding increases the number of offspring but decreases parental investment of Red Sea coral Stylophora pistillata
Source: Ecol Evol. 2019 Oct 2;9(21):12245–58. doi: 10.1002/ece3.5712 (PMC6854114; doi:10.1002/ece3.5712)
Supplement: Supplementary file 3 [file ECE3-9-12245-s003.pdf]

SI Table 2 Mean ± standard deviation relative abundance and concentration of individual fatty acids in adult coral tissue (*n* = 5), symbiont tissue (n = 5), *Artemia* coral feed (n = 3), and trough water (seawater (n = 1)). Blank cells inidicate the compound was not found. The FAs are abbreviated as Cx:y, where óxô is the number of carbons and óyô the number of double bonds. The first double bond position is counted from the methyl end of the lipid alkyl chain (n or ɤ nomenclature).

Compound number	Compound name	Adult coral host tissue				Symbiont tissue				Artemia nauplii				Seawater	
		µg FA/ g DW	st. dev.	Relative %	st. dev.	µg FA/ g DW	st. dev.	Relative %	st. dev.	µg FA/ mL feed	st. dev.	Relative %	st. dev.	µg FA/ L	Relative %
1	C12:0	0.18	0.40	0.11	0.15	0.32	0.44	0.25	0.07	0.04	0.04	0.03	0.02	0.04	4.90
2	C14:0	5.82	3.94	1.64	0.21	9.51	3.40	3.63	1.45	1.60	0.45	1.24	0.09	0.09	9.29
3	C14:1*n*-7					0.56	1.25	0.40	0.00	0.08	0.03	0.06	0.01		
4	C14:1*n*-4	0.18	0.41	0.22	0.00	0.67	0.93	0.53	0.15	0.58	0.21	0.44	0.01		
5	12-Me C14:0									0.23	0.09	0.18	0.02		
6	13-Me C14:0									0.09	0.04	0.07	0.00		
7	C15:0					0.12	0.28	0.21	0.00	0.18	0.09	0.13	0.00	0.01	1.58
8	13-Me C15:0	4.47	2.39	1.31	0.27					0.12	0.07	0.09	0.01		
9	14-Me C15:0	3.50	1.72	1.05	0.21					0.39	0.16	0.29	0.02		
10	C16:0	70.06	56.68	18.25	1.64	55.67	29.01	19.34	3.68	24.12	7.35	18.95	3.69	0.37	40.50
11	C16:1*n*-9	2.66	1.63	0.83	0.37	0.47	0.65	0.37	0.13	0.48	0.19	0.36	0.05	0.01	1.33
12	C16:1n-7	11.02	9.18	2.93	0.41	15.25	6.31	5.57	1.46	1.34	0.50	1.02	0.11	0.03	2.77
13	14-Me C16:0									0.51	0.22	0.38	0.09		
14	15-Me C16:0									0.85	0.34	0.64	0.08		
15	C16:2*n*-6	1.14	1.39	0.51	0.11	4.53	1.80	1.79	0.70						
16	C17:0	0.15	0.34	0.38	0.00	0.15	0.34	0.26	0.00	0.65	0.34	0.47	0.15		
17	2-hexyl Cyclopropanoctanoic acid									0.56	0.23	0.42	0.04		
18	C16:3*n*-4	7.90	7.54	1.90	1.30	2.35	2.15	0.99	0.37						1.07
19	C18:0	59.65	32.52	17.44	2.63	16.48	5.92	6.25	1.97	6.05	2.46	4.54	0.58	0.27	28.94
20	C18:1*n*-9	19.14	22.58	4.41	1.84	10.13	6.12	3.52	1.16	19.50	6.23	15.04	0.75	0.03	3.26
21	C18:1*n*-7/*n*-4	9.39	10.18	2.27	0.70	2.88	2.83	1.17	0.49	6.45	2.79	4.81	0.81	0.02	2.17
22	C18:2*n*-9									0.43	0.16	0.32	0.01		
23	C18:2*n*-6	2.55	2.44	0.78	0.12	0.85	1.22	0.63	0.13	4.94	1.96	3.72	0.31		
24	2-octyl Cyclopropanoctanoic acid					0.67	1.49	0.48	0.00	0.40	0.08	0.32	0.10	0.01	1.04
25	C18:3*n*-6 or C16:4*n*-3	2.81	3.04	0.78	0.19	6.06	5.99	2.08	0.70	0.29	0.23	0.19	0.14		
26	C18:3*n*-3									30.66		23.37	1.44		
27	C20:0	1.98	2.20	0.57	0.14	32.12	71.35	11.68	16.19	0.15	11.51	0.11	0.10	0.01	1.37
28	C18:4*n*-3	11.80	7.10	3.34	0.65	87.82	51.74	28.97	3.62	3.05	0.16	2.30	0.24		
29	C20:1*n*-11/*n*-7	4.19	6.07	1.04	0.60	0.99	1.42	0.55	0.41	0.58	1.21	0.43	0.13		
30	C20:2*n*-9/*n*-6	0.87	1.34	0.34	0.04	6.30	2.94	2.30	0.77	0.27	0.27	0.20	0.02		
31	C20:3*n*-6	4.74	7.28	1.10	0.73	1.86	1.83	0.76	0.31	0.18	0.16	0.14	0.01	0.02	1.76
32	C20:4*n*-6	56.33	31.46	16.30	2.11	11.28	6.19	3.87	0.89	3.02	0.12	2.28	0.34		
33	C20:3*n*-3?									1.09	1.15	0.81	0.23		
34	C20:4*n*-6									0.06	0.51	0.04	0.04		
35	22:00	4.59	6.25	1.01	0.58	0.95	1.47	0.70	0.30	0.76	0.07	0.57	0.12		
36	C20:5*n*-3	19.92	8.49	6.15	1.45	16.86	20.27	4.40	1.87	6.88	0.32	5.22	0.70		
37	C22:6*n*-9?	28.58	14.68	8.57	1.67	7.46	9.44	3.07	3.47	3.26	2.58	2.48	0.53		
38	C24:0	0.45	1.02	0.09	0.16					0.09	1.28	0.06	0.03		
39	C24:1*n*-9	5.68	3.08	1.66	0.30	1.76	1.83	0.67	0.14	0.28	0.08	0.21	0.10		
40	C22:6*n*-3	29.51	31.38	6.87	2.17	27.05	23.81	7.85	1.64	10.89	0.16	8.22	1.67		
	**SUM**	**370.47**	**267.42**			**321.33**	**231.12**			**131.01**	**4.42**			**0.91**	
